# Fabrication of hollow flower-like magnetic Fe_3_O_4_/C/MnO_2_/C_3_N_4_ composite with enhanced photocatalytic activity

**DOI:** 10.1038/s41598-021-81974-2

**Published:** 2021-01-28

**Authors:** Mingliang Ma, Yuying Yang, Yan Chen, Jiabin Jiang, Yong Ma, Zunfa Wang, Weibo Huang, Shasha Wang, Mingqing Liu, Dongxue Ma, Xiaoning Yan

**Affiliations:** 1 School of Civil Engineering, Qingdao University of Technology, Qingdao, 266033 People’s Republic of China; 2 School of Material Science and Engineering, Shandong University of Science and Technology, Qingdao, 266590 People’s Republic of China; 3 Key Laboratory of Marine Environmental Corrosion and Bio-Fouling, Institute of Oceanology, Chinese Academy of Sciences, Qingdao, 266071 People’s Republic of China; 4 School of Environmental and Municipal Engineering, Qingdao University of Technology, Qingdao, 266033 People’s Republic of China

**Keywords:** Environmental sciences, Materials science, Nanoscience and technology

## Abstract

The serious problems of environmental pollution and energy shortage have pushed the green economy photocatalysis technology to the forefront of research. Therefore, the development of an efficient and environmentally friendly photocatalyst has become a hotpot. In this work, magnetic Fe_3_O_4_/C/MnO_2_/C_3_N_4_ composite as photocatalyst was synthesized by combining in situ coating with low-temperature reassembling of CN precursors. Morphology and structure characterization showed that the composite photocatalyst has a hollow core–shell flower-like structure. In the composite, the magnetic Fe_3_O_4_ core was convenient for magnetic separation and recovery. The introduction of conductive C layer could avoid recombining photo-generated electrons and holes effectively. Ultra-thin g-C_3_N_4_ layer could fully contact with coupled semiconductor. A Z-type heterojunction between g-C_3_N_4_ and flower-like MnO_2_ was constructed to improve photocatalytic performance. Under the simulated visible light, 15 wt% photocatalyst exhibited 94.11% degradation efficiency in 140 min for degrading methyl orange and good recyclability in the cycle experiment.

## Introduction

In recent years, with the increase of wastewater discharge, a large amount of toxic and harmful organic pollutants are put into the water, which are difficult to completely degrade^1–6^. Advanced photocatalytic oxidation technology with strong oxidation capacity and high efficiency was considered as a very promising wastewater treatment means for the degradation of organic pollutants^7–14^. Among them, semiconductor-based photocatalysts with mild reaction conditions were most widely used^15–22^. In addition, various styles of self-assembled photocatalyst nanostructures were synthesized, such as one-dimensional nanotubes^23^, two-dimensional layered structure^24,25^, three-dimensional network structure^26^, three-dimensional flower-like structure^27^ and etc.

MnO_2_ has some advantages including low cost, high stability and environmental friendliness. Besieds, its narrow band gap can increase the utilization of visible light, which is very promising as a photocatalyst^28–32^. The layered structure of δ-MnO_2_ is formed by the MnO_6_ octahedral layer with shared edges, and there are some cations and H_2_O molecules between the layers to maintain the charge balance^33^. δ-MnO_2_ has been widely used as a catalyst to purify the environment^34–36^. However, while the narrow band gap increases the usage of visible light, it is also accompanied by the rapid recombination of photo-generated electrons and holes. The low photocatalytic efficiency restricts pure MnO_2_ for practical applications^37–39^.

Coupling MnO_2_ with other semiconductors to build a heterojunction is an effective method to prevent the recombination of photo-generated carriers. Wang et al.^40^ first reported that g-C_3_N_4_ could decompose water to generate hydrogen by visible light irradiation. Subsequently, owing to excellent visible light activity and chemical stability, g-C_3_N_4_ as photocatalyst has attracted a lot of attention^41–43^. Recently, Zhu et al.^44^ coated g-C_3_N_4_ on the semiconductor surface to form an ultra-thin g-C_3_N_4_ layer, avoiding the low contact rate between the bulk g-C_3_N_4_ and the coupling semiconductor interface. Therefore, the design of the composite with ultra-thin g-C_3_N_4_ layer and MnO_2_ is expected to achieve significant charge transfer at their interface by facilitating the separation of photo-generated carriers, thereby enhancing photocatalytic activity.

**Figure 1:**
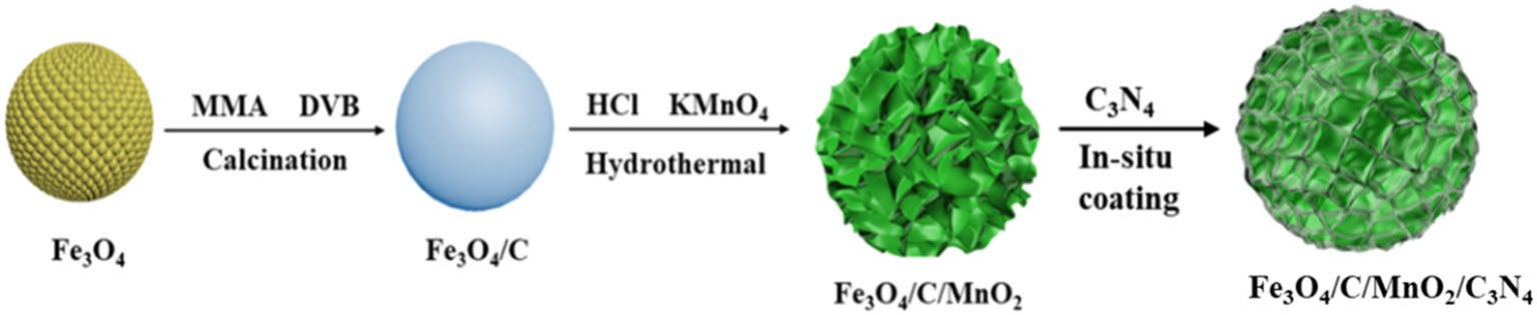
Fabrication of magnetic flower-like Fe_3_O_4_/C/MnO_2_/C_3_N_4_ photocatalyst.

In our previous research, Fe_3_O_4_ was introduced as a magnetic core to construct a magnetically recyclable core–shell structure photocatalyst. The existence of magnetic core was beneficial to the recycle of the catalyst^45,46^. In this paper, hollow Fe_3_O_4_ microspheres were also synthesized by using a hydrothermal method as magnetic cores. Subsequently, the carbon layer was obtained by calcining the polymer layer formed on the periphery of Fe_3_O_4_ microspheres, which can protect the core and act as an electronic conductor simultaneously. Then, flower-like MnO_2_ was grown on the periphery of Fe_3_O_4_/C microspheres through the hydrothermal method. Finally, the transparent CN precursor produced with the aid of the neutral hydrothermal process was polymerized in situ on the surface of above microspheres at a low temperature. A flower-like Fe_3_O_4_/C/MnO_2_/C_3_N_4_ composite photocatalyst with core–shell structure was obtained. The photocatalyst displayed remarkable photocatalytic activity for degrading the organic dye methyl orange (MO). The specific synthetic steps are shown in Fig. 1.

## Experimental section

### Preparation of Fe_3_O_4_/C core–shell composite

First, hollow Fe_3_O_4_ microspheres were obtained by the hydrothermal method^47^. Then the polymer layer was prepared through a distillation precipitation process. 0.10 g Fe_3_O_4_ microspheres were ultrasonically dispersed in 80 mL acetonitrile. 1.0 mL divinylbenzene (DVB), 1.0 mL methyl methacrylate (MMA), and 0.040 g 2,2-azobisisobutyronitrile (AIBN) were also added into the above solution. The system was heated in 90 °C water bath for 2 h. Afterwards, Fe_3_O_4_/P(MMA-DVB) microspheres were obtained by using an external magnet, and washed three times. Then, the Fe_3_O_4_/P(MMA-DVB) sample was calcined at 600 °C for 2.0 h to obtain Fe_3_O_4_/C microspheres.

### Preparation of Fe_3_O_4_/C/MnO_2_ flower-like composite

0.30 g Fe_3_O_4_/C microspheres were fully dispersed to 80 mL 0.055 M KMnO_4_ solution. Next, 1.0 mL HCl was added to the mixture dropwise. Thereafter, the mixture was transferred to an autoclave and heated to 100 °C for 6.0 h. At last, flower-like Fe_3_O_4_/C/MnO_2_ microspheres with core–shell structure were obtained by using an external magnet, and washed three times and lyophilized.

### Preparation of Fe_3_O_4_/C/MnO_2_/C_3_N_4_ composite

6.0 g dicyandiamide was calcined at 550 °C for 4.0 h to produce g-C_3_N_4_. Thereafter, 2.0 g g-C_3_N_4_ powder was dispersed in 80 mL deionized water, and then heated at 210 °C for 6 h to form a CN transparent precursor. Fe_3_O_4_/C/MnO_2_ microspheres were added to the precursor (5.0, 10, 15, 20, 30 wt%). The solvent was slowly removed through a lyophilized process. Finally, Fe_3_O_4_/C/MnO_2_/C_3_N_4_ flower-like photocatalyst was obtained via annealing at 200 °C for 4.0 h in a tube furnace under N_2_ protection.

### Characterization

Scanning electron microscope (SEM, JSM-6700F, JEOL Ltd., Japan) was employed to obtain a surface topography image of the samples. Transmission images were gotten by using a high-resolution transmission electron microscope (TEM, JEM-3010, Hitachi Co., Japan). X-ray diffraction patterns of samples were obtained by the use of an X-ray diffractometer (XRD, Shimadzu XRD-7000, Shimadzu Co., Japan). X-ray photoelectron spectrometer (XPS, JPS-9010 MC, JEOL Ltd., Japan) was utilized to obtain the samples’ surface elemental composition of the samples. Brunauer–Emmett–Teller (BET, ASAP 2020, Quantachrome, US) means was used to test the pore size and specific surface area of the catalyst. The saturation magnetization of the samples was obtained by employing a vibrating sample magnetometer (VSM, Lake Shore 7307, Lake Shore Ltd., USA). A photochemical reactor (BL-GHX-V, Shanghai Bilang Instruments Co., Ltd., China) was used to simulate the illumination. The ultraviolet–visible absorption spectra were measured on an ultraviolet–visible spectrophotometer (UV–vis, UV-5200PC, YuanXi, China).

### Photocatalytic experiment

Firstly, 20 mg Fe_3_O_4_/C/MnO_2_/C_3_N_4_ photocatalyst were added to 65 mL, 10 mg/L MO solution. Under dark environment, the mixture was agitated to reach adsorbed-desorbed equilibrium. Secondly, photocatalytic reaction was carried out with simulate light stemming from a 400 W metal halide lamp. The absorbance of the solution at intervals was monitored with the help of UV–visible spectrophotometer. Ultimately, the degradation curves of the MO solution were recorded, followed by the calculation of photocatalytic degradation rate.

## Results and discussion

**Figure 2:**
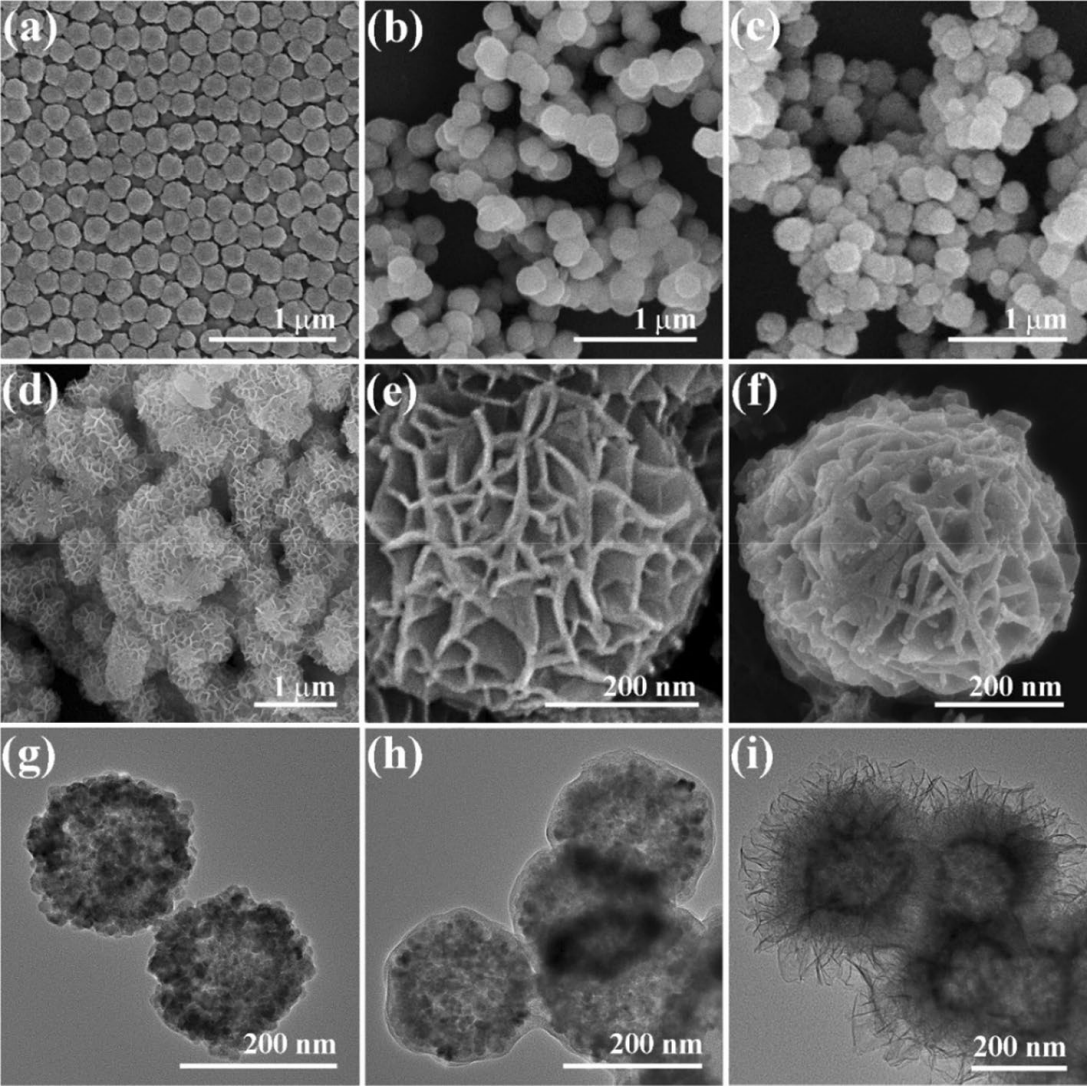
SEM images of hollow Fe_3_O_4_ microspheres (**a**), Fe_3_O_4_/P(MMA-DVB) microspheres (**b**), Fe_3_O_4_/C microspheres (**c**), Fe_3_O_4_/C/MnO_2_ flower-like microspheres (**d**,**e**), and Fe_3_O_4_/C/MnO_2_/C_3_N_4_ flower-like microspheres (**f**), TEM images of hollow Fe_3_O_4_ microspheres (**g**), Fe_3_O_4_/C core–shell microspheres (**h**) and flower-like Fe_3_O_4_/C/MnO_2_/C_3_N_4_ microspheres (**i**).

SEM and TEM images of samples are shown in Fig. 2. In Fig. 2a, Fe_3_O_4_ microspheres prepared by the hydrothermal method have good dispersibility and uniform size of about 200 nm. Figure 2b shows Fe_3_O_4_/P(MMA-DVB) microspheres prepared by distillation precipitation process. Compared with the former, the surface of the latter becomes much smoother, which proves the successful formation of polymer coating. And these polymer core–shell microspheres have a diameter of 225 nm. To obtain the conductive carbon layer, the polymer microspheres were calcined and carbonized. The SEM image of Fe_3_O_4_/C microspheres is displayed in Fig. 2c. One can see that the original core–shell structure of the material is not destroyed after the calcination treatment. And the agglomeration that originally occurred in Fe_3_O_4_/P(MMA-DVB) polymer microspheres has been slightly weakened due to the carbonization treatment. From Fig. 2d,e, it can be found out that the flower-like morphology of the composite microspheres produced by the hydrothermal method is composed of MnO_2_ intersecting sheets. And the overall particle size is about 480 nm. As shown in Fig. 2f, the overall flower-like morphology has not changed, but the thickness of the MnO_2_ flower sheets has increased significantly. This case indicates that the ultra-thin C_3_N_4_ layer is successfully formed on the surface of MnO_2_ to form a flower-like Fe_3_O_4_/C/MnO_2_/C_3_N_4_ composite photocatalyst. It can be seen from Fig. 2g that the synthesized magnetic microspheres have a clear hollow structure with a particle size of about 200 nm. Figure 2h shows the TEM image of the Fe_3_O_4_/C microspheres, which have a core–shell structure with 13 nm thickness of C shell. Figure 2i is the TEM image of the flower-like Fe_3_O_4_/C/MnO_2_/C_3_N_4_ microspheres. It can be found out that the composite photocatalyst with a complete magnetic core and flower-like shell exhibits the diameter of around 480 nm. According to these results, the composite photocatalyst with a magnetic core and flower-like shell was successfully prepared.

**Figure 3:**
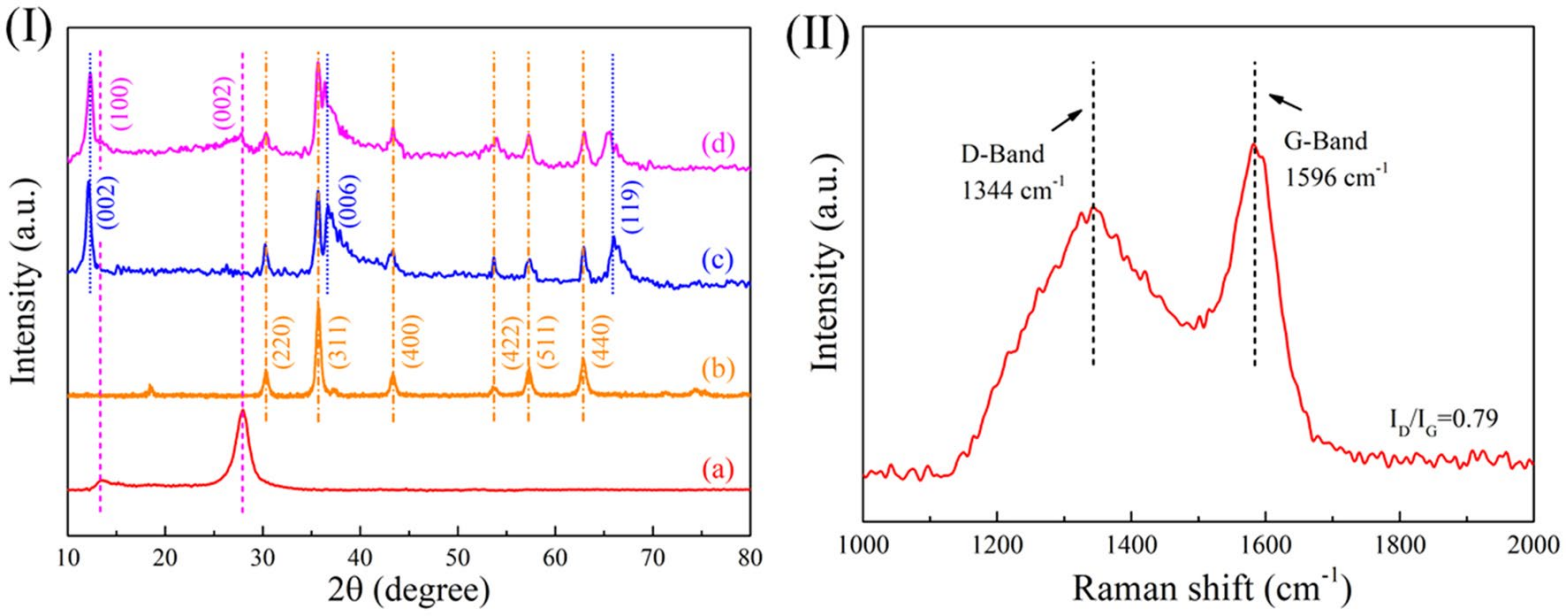
(**I**) XRD patterns of pure g-C_3_N_4_ (**a**), Fe_3_O_4_ microspheres (**b**), Fe_3_O_4_/C/MnO_2_ flower-like microspheres (**c**) and Fe_3_O_4_/C/MnO_2_/C_3_N_4_ flower-like microspheres (**d**). (**II**): Raman spectrum of Fe_3_O_4_/C microspheres.

The crystal phase composition of the composite was demonstrated by XRD characterization, as shown in Fig. 3I. Figure 3I-a is the diffraction curve of the bulk g-C_3_N_4_ obtained by pyrolysis of dicyandiamide. The strong peak near 27.4° belongs to the (002) plane, corresponding to the crystal plane stack of the CN aromatic system^48^. The broad peak at 13.0° belongs to the (100) plane ascribed to the triazine repeat unit^44^. Figure 3I-b is the diffraction curve of Fe_3_O_4_ microspheres showing the diffraction peaks at 30.12°, 35.41°, 43.10°, 53.43°, 57.11°, and 62.52°, which are attributed to (220), (311), (400), (422), (511) and (440) crystal planes of Fe_3_O_4_ (JCDPS 85-1436)^49^. The sharp peaks indicate that the synthesized Fe_3_O_4_ are well crystallized. In Fig. 3I-c, in addition to the diffraction peaks of Fe_3_O_4_ component, the diffraction peaks at 12.20°, 36.70°, and 65.70° correspond to (002), (006), and (119) crystal planes, in consistence with the crystal planes of MnO_2_ (JCDPS 18-0802)^50^. This case indicates that the MnO_2_ is successfully synthesized. The diffraction curve of the Fe_3_O_4_/C/MnO_2_/C_3_N_4_ photocatalyst in Fig. 3I-d reveals that except for the diffraction peaks of Fe_3_O_4_ and MnO_2_, the diffraction peaks appear at 13.00° and 27.40° are separately assigned to the (100) and (002) crystal planes of g-C_3_N_4_. This situation indicates that CN precursor successfully becomes g-C_3_N_4_ after low temperature polymerization and high temperature calcination. XRD results show that the Fe_3_O_4_/C/MnO_2_/C_3_N_4_ composite photocatalyst were successfully synthesized.

The XRD patterns cannot verify the existence of the C layer. For further confirming the formation of the C layer, the Raman test was used to characterize the Fe_3_O_4_/C sample. The spectrum in Fig. 3II indicates two different peaks at 1344 cm^−1^ and 1596 cm^−1^, corresponding to D-band and G-band of carbon material, respectively. These results confirm the carbonization of Fe_3_O_4_/P(MMA-DVB) material, and Fe_3_O_4_/C microspheres are successfully obtained. These two bands are related to the A_1g_ phonon of *sp*^3^ carbon atoms in disordered graphite and the in-plane vibration of *sp*^2^ carbon atoms in the crystalline graphite, respectively^51^. The peak intensity ratio (I_D_/I_G_) can evaluate the carbon material’s crystallinity. The smaller the value is, the higher the degree of atomic order is^52^. Herein, the value is 0.79, meaning that the carbon material is graphitized partially. Therefore, the presence of the carbon matrix can improve the electronic conductivity and help avoid the recombination of photo-generated electron holes.

**Figure 4:**
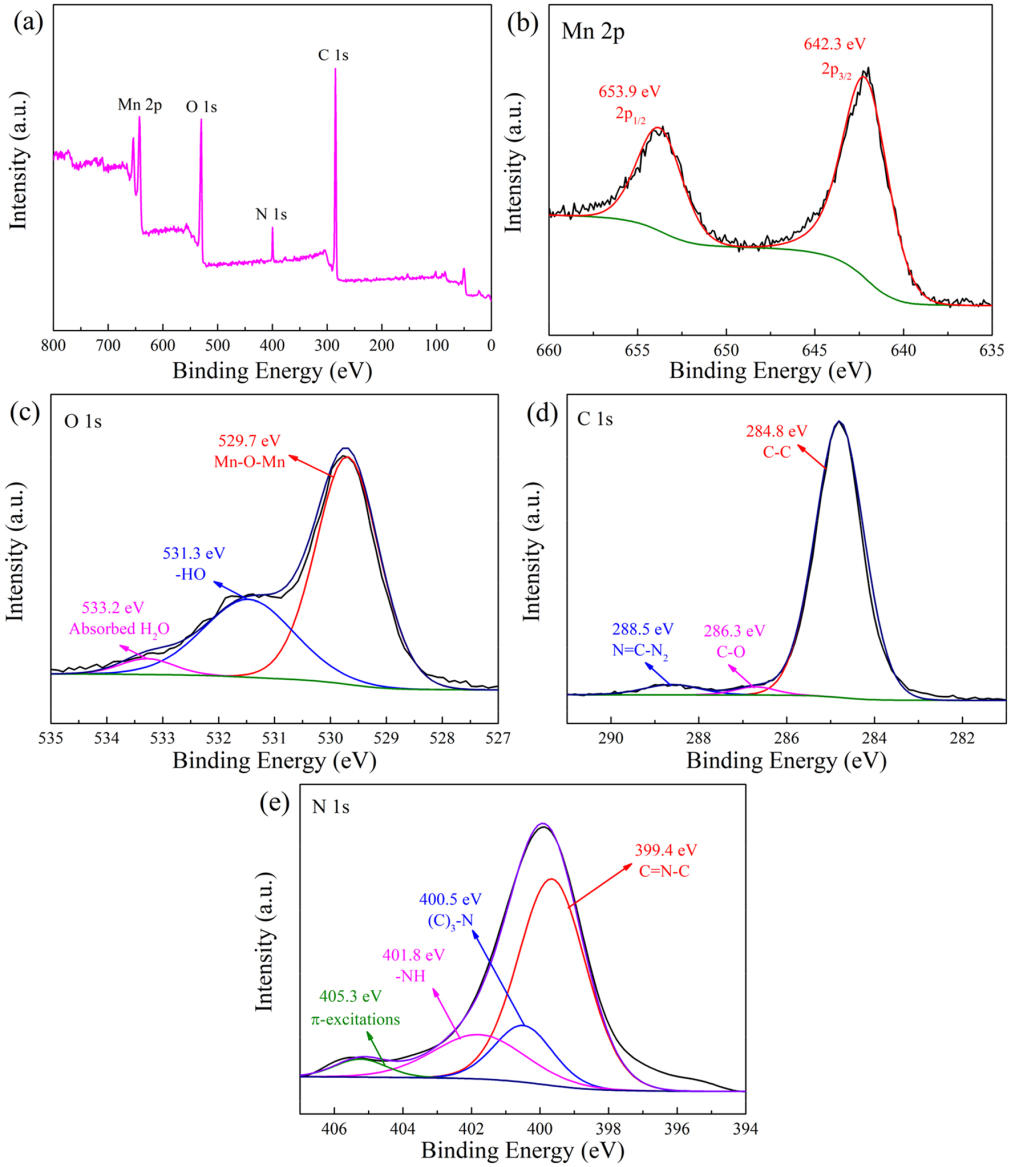
XPS spectra of Fe_3_O_4_/C/MnO_2_/C_3_N_4_ photocatalyst (**a**), Mn 2*p* (**b**), O1*s* (**c**), C1*s* (**d**) and N1*s* (**e**).

The surface chemical composition and the chemical state of the products were demonstrated by XPS characterization. Figure 4a is the full-scan spectrum of the photocatalyst, presenting the peaks of Mn, O, N, and C elements. From Fig. 4b, as for the Mn 2*p* spectrum, two peaks at 653.9 eV and 642.3 eV correspond to Mn 2*p*_1/2_ and Mn 2*p*_3/2_. With respect to the O1*s*, as illustrated in Fig. 4c, three peaks at 529.7 eV, 531.3 eV, 533.2 eV are fitted, which are separately attributed to the Mn–O–Mn lattice oxygen, surface hydroxyl and surface adsorbed oxygen. The C1*s* spectrum in Fig. 4d shows the sub-bands centered at 284.8 eV and 288.5 eV, which are ascribed to the C–C coordination of the surface-unstable carbon and N=C–N_2_ of g-C_3_N_4_. In addition, there is another peak centering at 286.3 eV, which is assigned to the C-O bond formed between the C of C_3_N_4_ and the O of MnO_2_. This result indicates that MnO_2_ and g-C_3_N_4_ are closely connected and form a solid MnO_2_/g-C_3_N_4_ interface, promoting the transfer and separation of photo-generated carriers. In the case of the N1*s* spectrum (Fig. 4e), the peaks at 399.4 eV, 400.5 eV, 401.8 eV, and 405.3 eV are separately designated as carbon-bonded *sp*^2^ hybrid aromatic C=N–C, a tertiary nitrogen bonded to a carbon atom in the form N–(C)_3_, NH and the charge effect or positive charge localization in the heterocyclic ring. The XPS spectra powerfully verify the surface chemical composition of the Fe_3_O_4_/C/MnO_2_/C_3_N_4_ photocatalyst.

The specific surface area and the pore structure of Fe_3_O_4_, Fe_3_O_4_/C, Fe_3_O_4_/C/MnO_2_ and Fe_3_O_4_/C/MnO_2_/C_3_N_4_ products are listed in Table 1. The former of the Fe_3_O_4_/C/MnO_2_ and Fe_3_O_4_/C/MnO_2_/C_3_N_4_ products are 119.56 m^2^/g and 120.25 m^2^/g, and the latter of them are 0.35 cm^3^/g and 0.31 cm^3^/g. Since C_3_N_4_ does not significantly affect the morphology of the composite structure, these parameters of the two samples are almost similar. The higher values are owing to the flower-like structure of the composite photocatalyst. The increase in specific surface area is conducive to exposing more active sites and increasing more surface adsorption, followed by improving catalytic performance.

**Table 1:** Specific surface area and pore parameters of samples.

Entry	Sample	Surface area (m^2^/g)	Pore width (nm)	Pore volume (cm^3^/g)
1	Fe_3_O_4_	38.47	4.01	0.17
2	Fe_3_O_4_/C	59.87	2.23	0.21
3	Fe_3_O_4_/C/MnO_2_	119.56	6.94	0.35
4	Fe_3_O_4_/C/MnO_2_/C_3_N_4_	120.25	6.35	0.31

**Figure 5:**
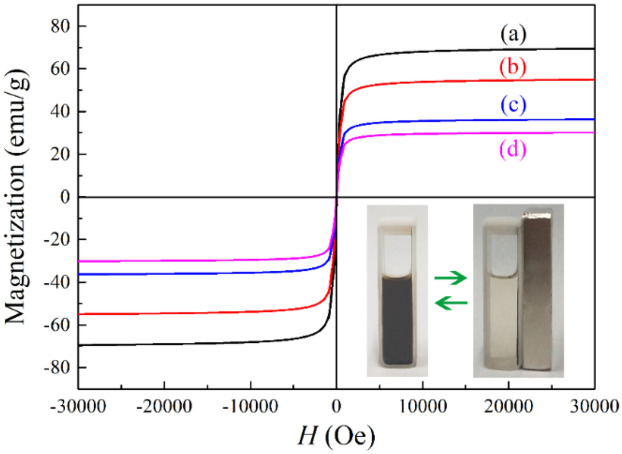
Hysteresis loop spectra of Fe_3_O_4_ microspheres (**a**), Fe_3_O_4_/C core–shell microspheres (**b**), Fe_3_O_4_/C/MnO_2_ flower-like microspheres (**c**) and flower-like Fe_3_O_4_/C/MnO_2_/C_3_N_4_ photocatalyst (**d**).

To evaluate the saturation magnetization value of Fe_3_O_4_, Fe_3_O_4_/C, Fe_3_O_4_/C/MnO_2_ and Fe_3_O_4_/C/MnO_2_/C_3_N_4_, VSM measurement is conducted. It can be seen from Fig. 5a that the magnetization value of the Fe_3_O_4_ microspheres is 70.58 emu/g. After the carbon layer is recombined, the value of Fe_3_O_4_/C microspheres decreases to 56.97 emu/g (Fig. 5b). After the flower-like MnO_2_ was fabricated, the content of Fe_3_O_4_ component is decreasing, which leads to the value of Fe_3_O_4_/C/MnO_2_ microspheres decreases obviously to 37.62 emu/g (Fig. 5c). With the further formation of g-C_3_N_4_, the value is 30.02 emu/g (Fig. 5d). This value still meets the needs of magnetic separation. As shown in the illustration, when the magnet is placed next to the Fe_3_O_4_/C/MnO_2_/C_3_N_4_ photocatalyst suspension, the photocatalyst can be quickly attracted to the side of the cuvette in a short time. The results show that the photocatalyst has a good magnetic response to the magnetic field, favoring the magnetic separation from the mixed solution.

**Figure 6:**
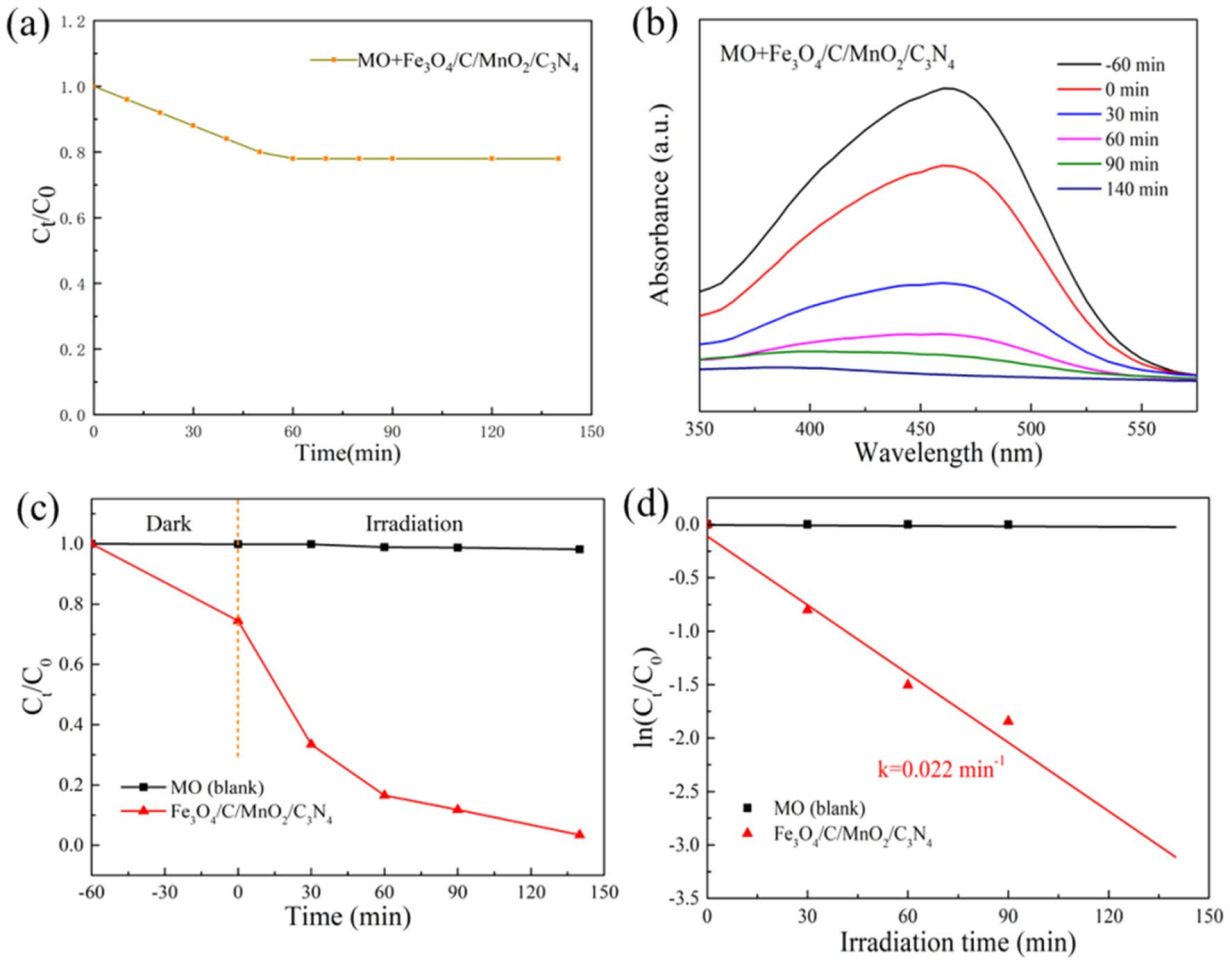
Adsorption of MO on sample in dark reaction (**a**), UV–vis spectra of MO solution at different times in the presence of Fe_3_O_4_/C/MnO_2_/C_3_N_4_ photocatalyst (**b**), C_t_/C_0_ change with time (**c**), first-order kinetic model linear fit (**d**). Reaction conditions: [MO] = 65 mL, 10 mg/L, [photocatalyst] = 20 mg, [light] = 400 W metal halide lamp, T = 25 °C, t = 140 min.

Determining the adsorption capacity of the photocatalyst in dark reaction, then degrading MO under simulated light is used to investigate the photocatalytic activity of the prepared photocatalyst, and the results are shown in Fig. 6. Figure 6a reveals the mixture reached adsorption–desorption equilibrium within 60 min. And Fe_3_O_4_/C/MnO_2_/C_3_N_4_ can adsorb about 22% of MO within 60 min, which is related to its higher specific surface area (120.25 m^2^/g). Figure 6b displays that UV–Vis is employed to monitor the change in the absorbance of the solution during the photocatalytic reaction. In Fig. 6b, one can clearly view that MO was almost completely degraded with adding Fe_3_O_4_/C/MnO_2_/C_3_N_4_ composite photocatalyst after 140 min. The photocatalytic degradation MO over Fe_3_O_4_/C/MnO_2_/C_3_N_4_ could be described by the following reactions:

1 $${\text{photocatalyst}} + hv \to {\text{photocatalyst}}\left( {e^{ - } } \right) + {\text{photocatalyst}}\left( {h^{ + } } \right)$$

2 $${\text{H}}_{2} {\text{O}} + h^{ + } \to ^{ \cdot } {\text{OH}} + {\text{H}}^{ + }$$

3 $${\text{O}}_{2} + e^{ - } \to ^{ \cdot } {\text{O}}_{2}^{ - }$$

4 $${\text{MO}} +^{ \cdot } {\text{OH/O}}_{2}^{ - } \to {\text{Mineralisation}}\;{\text{products}}$$

Figure 6c indicates the change of the MO concentration ratio C_t_/C_0_ with varying the light time, in which C_0_ and C_t_ are the initial concentration of MO and the concentration of MO during the reaction, respectively. The degradation rate of MO solution with Fe_3_O_4_/C/MnO_2_/C_3_N_4_ photocatalyst reaches 94.11%. From Fig. 6d, this reaction is attributed to a pseudo first-order reaction, which belongs to the Langmuir–Hinshelwood model with ln (C_t_/C_0_) = −*k*t. In the formula, *k* is the apparent first-order rate constant. The calculated rate constant *k* of Fe_3_O_4_/C/MnO_2_/C_3_N_4_ photocatalyst is 0.022 min^−1^. The excellent photocatalytic performance of Fe_3_O_4_/C/MnO_2_/C_3_N_4_ composite material benefits from the synergistic effect between the various components.

**Figure 7:**
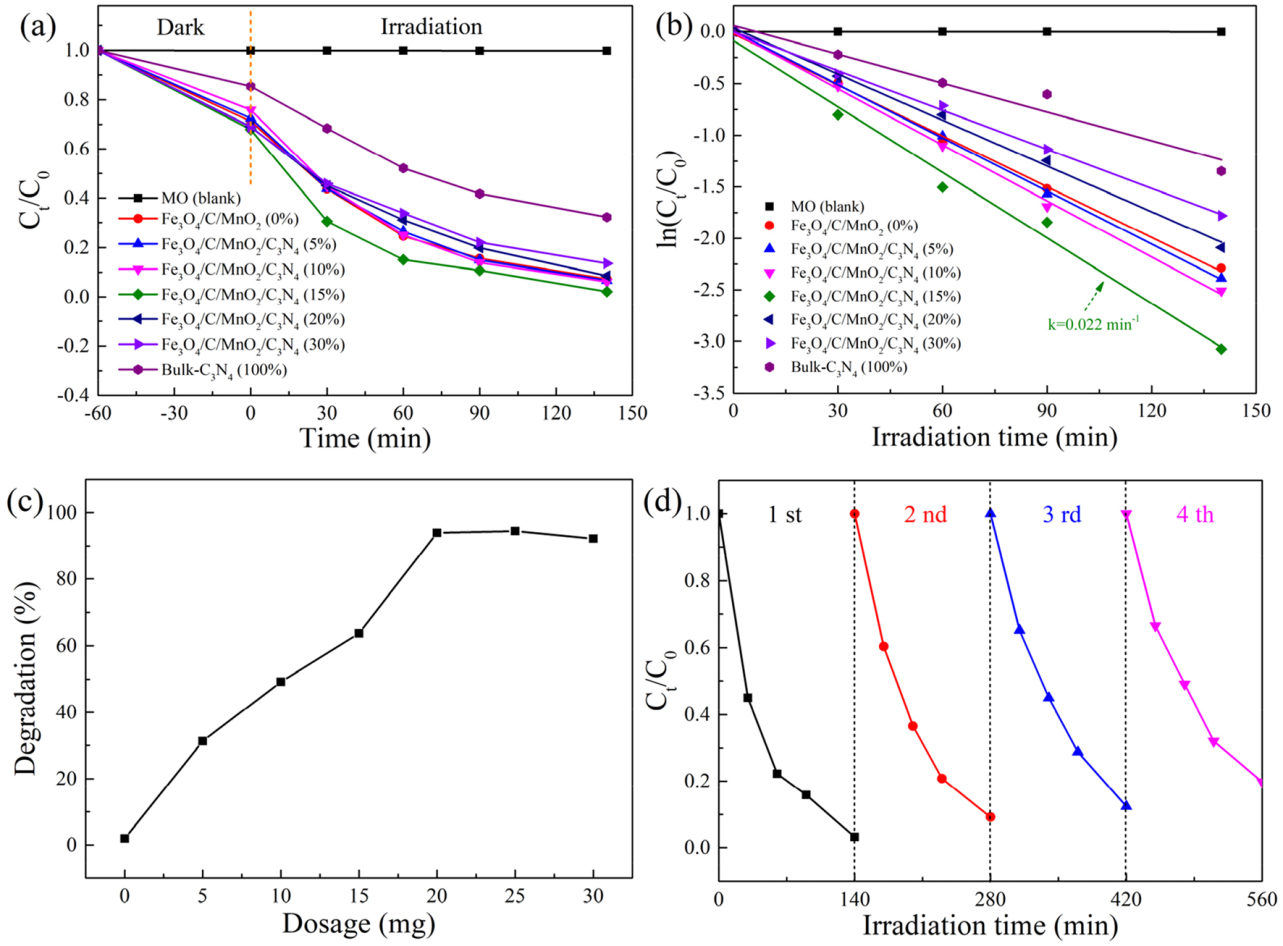
Effect of the amount of C_3_N_4_ on the degradation of MO (**a**,**b**), effect of the amount of Fe_3_O_4_/C/MnO_2_/C_3_N_4_ photocatalyst on the degradation of MO (**c**), cyclic test of MO degradation of Fe_3_O_4_/C/MnO_2_/C_3_N_4_ photocatalyst (**d**). Reaction conditions: [MO] = 65 mL, 10 mg/L, [light] = 400 W metal halide lamp, T = 25 °C, t = 140 min. For (**a**,**b**,**d**), [photocatalyst] = 20 mg.

In order to find the optimal ratio, the effect of amount of g-C_3_N_4_ on the photocatalytic efficiency was investigated. Meanwhile, determining the minimum optimal amount of photocatalyst in practical applications is important to reduce the costs. The composite photocatalyst containing different amounts of g-C_3_N_4_ (5%, 10%, 15%, 20%, 30%) were used to degrade MO dyes under the same conditions. From Fig. 7a,b, when the amount of g-C_3_N_4_ is 15%, the Fe_3_O_4_/C/MnO_2_/C_3_N_4_ composite photocatalyst has the highest value. In Fig. 7c, the effect of the amount of photocatalyst on the degradation efficiency is examined. The results show that the photocatalytic efficiency gradually increases when the amount of photocatalyst increases in the range of 0–20 mg, due to the effective reaction area and the reactive site increase. When the amount of photocatalyst continues to increase, the photocatalytic efficiency does not change significantly, which may be caused by the particle agglomeration affecting the increase of active sites. Therefore, the optimal dosage of Fe_3_O_4_/C/MnO_2_/C_3_N_4_ photocatalyst is 20 mg. Considering the industrial application of Fe_3_O_4_/C/MnO_2_/C_3_N_4_ nanoparticles, it is essential to investigate the recyclability and stability of the photocatalyst. The Fe_3_O_4_/C/MnO_2_/C_3_N_4_ was reused four times to examine their performances. And Fig. 7d reveals the results that the degradation rates for the four cycles are 94.11%, 90.42%, 88.37% and 79.69%, respectively. There is no doubt that after the photocatalyst is recycled, the conversion rate will decrease, which might result from the loss of sample during the cycle. However, even after four cycles, the value still has 79.69% that might be related with the structure stability of the used photocatalysts, strongly demonstrating that the designed photocatalyst has excellent recyclability.

**Figure 8:**
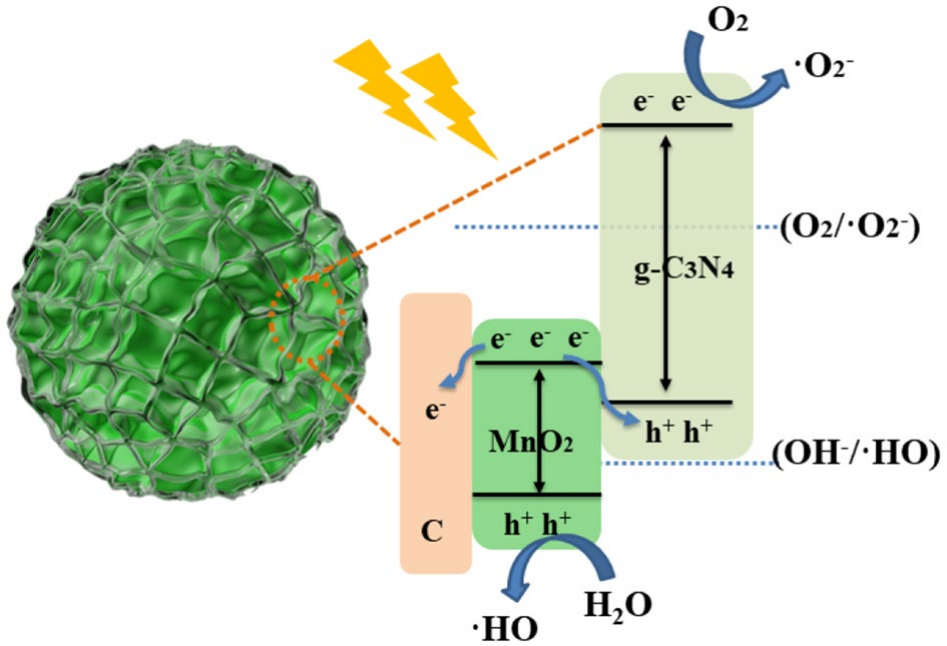
Schematic diagram of possible mechanism of Fe_3_O_4_/C/MnO_2_/C_3_N_4_ photocatalyst.

In this study, Fe_3_O_4_/C/MnO_2_/C_3_N_4_ photocatalyst was synthesized by compounding g-C_3_N_4_ on the surface of MnO_2_. In terms of enhanced photocatalytic activity, it is assumed that the charge transfer in the photocatalyst uses the Z-type mechanism, as shown in Fig. 8. For the individual g-C_3_N_4_ or MnO_2_ component, due to thermodynamic effects, photo-generated holes in g-C_3_N_4_ cannot oxidize OH^-^ to form •OH radicals, while photo-generated electrons in MnO_2_ cannot generate ^·^O_2_^−^ radicals effectively. Therefore, individual g-C_3_N_4_ or MnO_2_ material cannot possess good photocatalytic performances. However, after a heterojunction was fabricated between these two components, the photo-generated electrons in the conduction band of MnO_2_ can be transferred to the valence band of g-C_3_N_4_ and combined with the photo-generated holes there. This configuration of the Z-type scheme makes the utilization of holes from MnO_2_ and electrons from g-C_3_N_4_ remarkably enhanced. In addition, the conductive C layer can also increase the photo-generated electron–hole pairs’ separation in MnO_2_, which effectively prevents the recombination of photo-generated carriers. In the meantime, the higher specific surface area supplies much more active sites for photocatalytic activities. The prepared flower-like Fe_3_O_4_/C/MnO_2_/C_3_N_4_ photocatalyst forms a Z-type photocatalytic system, which effectively enhances the separation of carrier, so that the composite material has excellent photocatalytic degradation efficiency.

## Conclusions

In summary, a magnetic recyclable flower-like Fe_3_O_4_/C/MnO_2_/C_3_N_4_ heterojunction photocatalyst was prepared for degrading organic dyes. The Fe_3_O_4_ core was used to facilitate magnetic separation and recovery. The C layer could conduct photo-generated electrons in MnO_2_ and protect the core. The thin g-C_3_N_4_ layer was compounded on the surface of MnO_2_, which greatly improved the specific surface area and the reactive sites of the material. The obtained Fe_3_O_4_/C/MnO_2_/C_3_N_4_ composites exhibited enhanced photocatalytic performance for the degradation of MO solution (65 mL, 10 mg/L) under simulated light irradiation. The maximum photocatalytic degradation efficiency was 94.11% within 140 min. It was assumed that a Z-type heterojunction was fabricated between MnO_2_ and g-C_3_N_4_, which stimulated the electron transfer from the valence band of MnO_2_ to the conduction band of g-C_3_N_4_. This structure promoted the photo-generated electron–hole pairs’ separation, inhibited the free charges’ recombination, and improved effective use of visible light. In here, an effective method to construct heterostructure nanomaterials was provided for efficient photocatalytic degradation.
